# The Teeth of the Horse and Their Diseases

**Published:** 1883-04

**Authors:** R. Jennings


### Art. XV.—THE TEETH OF THE HORSE AND THEIR DISEASES.

BY R. JENNINGS, V.S.

*(Continued.)*

IT was my intention when I commenced writing upon this subject to have continued in each succeeding number of the Journal to the end, but circumstances, prevented my doing so. I trust, however, that your readers have not lost interest in the subject by the delay.

Let us go back forty years, when veterinary science in the Old World was yet in its infancy, and in the New the *farriers* were the *literàti*, to whose tender care its practice was intrusted. The title of veterinary surgeon was rarely heard in the United States. These were the days when symptoms of thoracic, or abdominal pain, in the horse, from whatever cause, were attributed to the presence of the then persecuted bot, and all sorts of nostrums were poured down the poor beast's throat or nostrils with a view to its destruction. Veterinary dentistry was then comparatively unknown. In fact it was not a subject for thought or investigation, and if by chance or accident the existence of a carious tooth in the horse's mouth was discovered it was looked upon as a very remarkable occurrence and believed to be an isolated case. Many a poor animal has been a silent sufferer for months and even years from the excruciating pain of an aching tooth, the symptoms indicating its presence, having been overlooked or attributed to other causes, and the poor animal brutally punished by an inhuman driver. My own investigations prove conclusively the frequent existence of this very common disease. It is a well known fact that caries of the teeth in the human family is the most common; a disease analogous to ulceration of bony tissue. It commences in the bony substance of the crown under its covering, the enamel. It is first observed as a minute brownish speck, usually upon the side, between or in front of the teeth and in the molars frequently upon the face of the tooth, extending inwards towards its centre, leaving the enamel a thin brittle shell, which from time to time is broken off, the roots remaining in the alveola comparatively sound. Not so in the horse· Caries of the teeth is confined with rare exceptions to the molar teeth. It is first observed in one or more of the indentations upon the face or grinding surface, confining its destructive agency almost exclusively to the crusta petrosa, a substance not found in the teeth of carnivorous animals, and constituting the bulk of the tooth; it fills up the intermediate spaces between and around the folds of the bone and the enamel, both of which dip deep into the crown of the molar teeth. Notwithstanding the rapid progress made in veterinary science during the last two decades, the expressed opinion of many owners of live stock in the country districts is that the teeth of the horse and other stock are not liable to decay. This very common error will be fully proven as we proceed. I made this the subject of a lecture delivered before the class at the Ohio State Agricultural College February 7th, 1855, a condensed report of which was published in the Ohio *Farmer*, of Cleveland. The subject at this time was a new one to the owners of horses, and to the veterinary profession as well.

At this early day I had in my collection some 350 well defined specimens of caries in the teeth of horses and mules, together with a number of specimens in the bovine animal. The symptoms of this disease in the teeth of the horse were generally associated with other diseases, or in some cases with vice; as is pleurisy and bronchitis, with pneumonia by a large number of persons professing veterinary knowledge at the present day. My attention was first called to the investigation of this subject in the year 1853. A horse condemned as glandered was sent to the knacker to be killed, which place I had been in the habit of visiting frequently for several years previous to this time. The peculiar appearance of the carcass of this animal led me to make a careful examination of the head, mouth and nostril, which convinced me that the trouble was not glanders, but the effect of a carious tooth, which the dissection of the parts proved to be the fourth molar in the upper jaw on the left side. This tooth had been almost entirely destroyed. The disease evidently commenced upon the face of the tooth, extending upwards between the folds of the bone and the enamel, continuing its course through the whole length of the fang, leaving but three ribbon like plates of bone and enamel indicating the great destruction going on and the terrible suffering the animal must have endured. Between these plates of bone, food had been from time to time forced up, finding a lodgment in the outrun of the superior maxillary bone, causing by its irritation the forming of an abscess, which found an outlet through the left nostril destroying the outer table of the maxillary bone, but not perforating the skin. I subsequently learned that this animal had been treated for chronic catarrh. The head and remains of the tooth, together with a large number of similar specimens of pathology, I sent to John Busteed, M.D., the founder of the New York College of Veterinary Surgeons, to be placed in the museum of that institution. The same year at the same place, in an examination of twenty-four heads of horses, I found the teeth in seventeen of them diseased, which in every case was confined to the upper molars. The bone and the enamel in the teeth of the horse appear to be almost impervious to the destructive agency of caries, or, at least, to resist its action for a long time. The attack is made upon the crusta petrosa, a softer substance than the bone, hence more easily injured; its more rapid wear preserves the grinding power of the molar teeth. When this third substance is attacked by the disease its destruction leaves the bone and enamel unprotected, in consequence of which, hard substances, as gravel stone, pieces of nail, &c., which sometimes finds its way into the food, coming in contact with these unprotected plates break them off, leaving cavities in the crown of the tooth, which may be detected by passing the fingers into the mouth through an adjustable gag. When these partitions remain firm it is difficult to detect the presence of the disease by such means, though its existence may be suspected from the fetid breath so common in these cases. Caries is by no means confined to the teeth of old horses; it is sometimes found in the permanent molars of young animals during the process of dentition. In the teeth of the old horse there are no thin plates of bone and enamel; the cavities are smooth and highly polished. This is due to the disappearance of these substances from the crown of the tooth, having been worn away from long use, or broken down in masticating the food. It is a singular fact that the fourth molar teeth in the upper jaw are the ones generally affected by this disease, on either or both sides of the jaw. The lower molars, the incisors and the canine teeth are rarely involved in this disease; probably not more than a score of such diseased teeth occur in the lower molars, and less than a dozen in the incisors, and not a single instance in the canine teeth has been discovered by the writer in more than five hundred cases of the disease in the teeth of the horse. In the year 1855 my attention was called to a horse belonging to the priest of St. Augustine's Church, Philadelphia. In this case the first molar tooth of the upper jaw right side was the tooth involved, which I extracted with a pair of forceps made for the purpose. A sinu had been formed opening into the nasal cavity. A solution of chloride of lime was used as a dressing, followed in a few days with tincture of myrrh; a perfect cure was the result. The same year I met a case of caries in the first and second molars of the under jaw on the left side. An abscess had formed at the roots of the teeth, causing considerable enlargement of the jaw bone in the form of spongy exostosis; the integument was perforated in many places, through which pus of a very fetid character was discharged. This animal was destroyed. When on a visit to Jackson, Michigan, in the year 1857, my attention was called to a bay mare belonging to the Ballard Bros., livery men, having a discharge from one side of the face about two inches below the eye, which had existed nearly two years. It had been treated for fistula without effect. On making an examination of the jaw I found the trouble arose from caries of the fourth molar tooth on the right side, which I extracted. In a few weeks the animal was well. In 1858 a horse was destroyed and examined at the dissecting room of the Veterinary College of Philadelphia, having an immense tumor extending over the entire surface of the posterior superior maxillary bone on the left side, passing inwards, filling up one nostril, and extending into the mouth. Autopsy revealed the entire destruction of the first molar tooth, and but a small fragment of the second remaining, having been almost entirely destroyed by caries. The above specimens were sent to New York with my collection. In 1859 the trotting horse Blue Dick, well-known in New York and Boston, was accidentally killed. The fourth molar tooth on either side of the upper jaw were well marked specimens of caries. Yet, as far as I could learn, he showed no symptoms indicating such a condition while living. During the winter of 1859-60 a brown mare, belonging to Mr. Andrew Godfrey, of Germantown, was sent to the clinic of the Philadelphia Veterinary College, having been pronounced glandered by a veterinary surgeon and ordered killed. Upon examination a large abscess was found opening into the nostril, caused by the presence of two carious teeth, the first and second molars of the right side. This mare was cast and placed under the influence of chloroform, and ten pieces of the carious teeth removed, the cavity cleaned out, and tow, saturated with tincture of myrrh, filled in, removing and dressing every day. Some four weeks ago this animal was sold for $150. Since my removal to the city of Detroit several interesting cases have occurred in my practice, some of which are worthy of mention, the pathological specimens of which I send you to be deposited in the museum of the Columbia Veterinary College.

No. 1, a black mare four years old, belonged to a Mr. Maux, of this city. This animal had a discharge of a fetid character from the left side of the face, some two inches anterior to the eye. It was treated by a professional veterinary dentist hailing from the city of New York, but he failing to benefit the animal, it was sold to Mr. Jas. Brown, then Superintendent of the Detroit Omnibus Company, who placed it in my charge for treatment. After a careful examination I discovered the cause to be caries of the fourth permanent molar tooth on the left side. The animal being young, and the crown of the tooth being almost entirely destroyed, the removal of the root was attended with considerable difficulty, but, with the assistance of Dr. J. Hawkins, I succeeded in its removal. What remained of the crown was pretty well broken up in its extraction, still it is a fine specimen of the disease. Mr. Brown removing from the city soon after, I lost sight of the animal, but subsequently learned that the wound healed nicely, leaving a very small denuded spot upon the cheek pointing out the former seat of discharge.

No. 2, a bay horse belonging to Dr. W. Dehn of this city, was a confirmed rearer and runaway. He died from congestion of the lungs, in September, 1880. This animal I did not see when living. An autopsy revealed the cause of death, and also the carious condition of the fourth upper molar tooth in the right upper jaw, with a deep cavity large enough to admit the end of a finger. The animal being of a very nervous temperament, such symptoms from such a cause might readily be associated with the suffering animal.

No. 3. Some two years ago Mr. Walton, a farmer residing near Grand Blank, Michigan, observed a bay mare munching her hay in an unnatural manner, but thought little of it. The mare gradually losing flesh, various preparations in the form of condition powders were given without benefit. A year passed by, the animal remaining still unthrifty. She now showed distress when feeding, particularly in eating hay. The breath was fetid, and gradually she became more emaciated. W. H. Green was called to see her, and, suspecting the cause, gave his opinion that the teeth were diseased. The owner was loth to believe the correctness of the diagnosis, never having supposed the teeth of a horse were subject to disease. I was sent for, and upon examination of the animal I found the inferior maxillary bone much enlarged and tumefied on the right side. I found the first molar of the under jaw ulcerated at its root, the second appearing to be involved. I decided on the removal of both, which was accomplished with little trouble. After the removal of these two teeth I found a third bony substance growing into the inferior maxillary bone, which caused the enlargement of the jaw. This, as a foreign body, I determined to remove as well, which I accomplished with much difficulty, breaking off its upper portion, which was lost. This, from its general appearance, might easily be mistaken for a double bodied incisor tooth, its fang articulating with the fang of the second molar tooth upon the outside growing from without upwards. This third or supernumary tooth was evidently the cause of the trouble.

No. 4. Hypertrophy of the fourth upper molar tooth of the right side. The animal from which this tooth was taken, died from marasmus, no doubt caused by long suffering from the presence of this tooth in the animal's mouth, full particulars of which I was unable to learn.

No. 5, a grey horse purchased by Mr. J. H. Maris, proprietor of one of the leading stables in this city, was observed champing his hay (the first day he entered the stable) in a very unusual manner, dropping it into the manger in quids, and eating grain very slowly, daintily, and with an unnatural lateral motion of the under jaw. My attention was at once called to the animal. From the above symptoms, in connection with a fetid breath, I suspected the cause of the trouble. On making an examination I discovered a very unusual wearing of the upper and lower fifth molar teeth on the right side of the jaws. The outer half of the upper molar tooth was completely destroyed by caries; the outer half of the opposing tooth in the under jaw, meeting with no resistance, was gradually filling up the cavity, thus reversing the wear upon the crown or face of the teeth, the upper molar wearing from within outwards and upwards, the lower molar from without inwards and downwards. While examining this tooth to test its firmness, the projecting inner portion broke off, leaving the root of the tooth still firmly planted in the jaw. As the animal was not a valuable one, and improved so much in feeding, I concluded not to risk an operation upon it, as under the circumstances its removal would require the trephining of the superior maxillary bone over the root of the tooth, and punching it out through the mouth, as it would be impossible to get the forceps securely upon the imprisoned root. I will endeavor to keep track of this animal, and should I hear of his death will secure the upper and lower jaw. These long years of experience have convinced me that caries of the upper molar teeth in horses, notwithstanding the present advanced state of veterinary dentistry, is far more common than is suspected by the most experienced veterinary surgeon. This, no doubt, is due to the difficulty of examining the molar teeth in the living animal, until the disease is so far advanced that the symptoms cannot be mistaken.

*(To be continued.)*

